# Stellate Ganglion Block to Increase Defibrillation Effectiveness in Acute Fulminant Myocarditis-Induced Refractory Ventricular Fibrillation

**DOI:** 10.1016/j.jaccas.2025.103752

**Published:** 2025-06-18

**Authors:** Arianna Morena, Carol Gravinese, Simone Frea, Filippo Angelini, Andrea Saglietto, Marinella Zanierato, Monica Andriani, Paolo Scacciatella, Gaetano Maria De Ferrari, Veronica Dusi

**Affiliations:** a Department of Medical Sciences, University of Turin, Turin, Italy; b Division of Cardiology, Cardiovascular and Thoracic Department, Città della Salute e della Scienza, Turin, Italy; c Department of Anesthesia, Intensive Care and Emergency, Città della Salute e della Scienza Hospital, Turin, Italy; d Division of Cardiology, Umberto Parini Regional Hospital, Aosta, Italy

**Keywords:** acute heart failure, cardiac magnetic resonance, ventricular fibrillation

## Abstract

**Background:**

Percutaneous left stellate ganglion block (PLSGB) is used to prevent ventricular arrhythmia recurrences in various settings. Clinical data on its impact on ventricular fibrillation (VF) defibrillation threshold are scant and limited to acute coronary syndromes.

**Case Summary:**

We present a case of refractory VF in acute fulminant myocarditis. Despite multiple drugs and hemodynamic mechanical support, the VF was unresponsive to several DC shock attempts, including double sequential and vector change defibrillation. PLSGB allowed stable sinus rhythm restoration.

**Discussion:**

PLSGB was performed to enhance the success of DC shock, highlighting the importance of acute neuromodulation strategies not only to prevent ventricular arrhythmia recurrences but also to improve defibrillation effectiveness. Our case is the first reported PLSGB usage in acute fulminant myocarditis-induced refractory VF.

## Visual Summary

**Figure:**
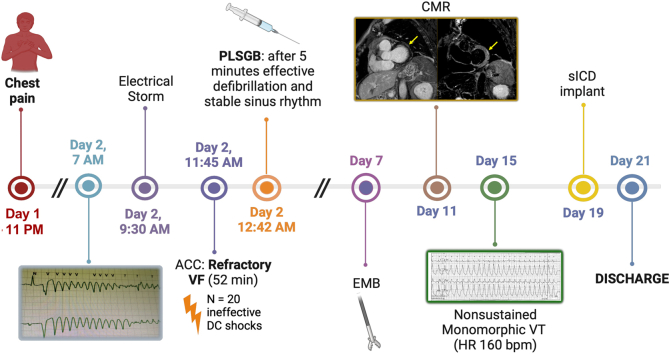

## History of Presentation

A 57-year-old man (weight: 90 kg) with a negative family history, hypertension, and no prior cardiac history presented to a spoke emergency department with typical chest pain. He also reported experiencing gastroenteritis, diarrhea, and fever in the past week. His physical examination was unremarkable; however, his electrocardiogram showed a heart rate of 55 beats/min with a 1-mm inferolateral ST-segment elevation. Transthoracic echocardiogram (TTE) revealed a mildly reduced left ventricular ejection fraction (LVEF) with hypokinesis of the mid-anterolateral segment. The initial laboratory tests indicated elevated high-sensitivity troponin T levels (150 ng/L; range: 0-14 ng/L), C-reactive protein (76 mg/dL), and white blood cells (17,700 cells/μL), with low potassium (3.1 mEq/L).

**Take-Home Messages**

• Beyond reducing the risk of VA recurrence, PLSGB can also be used to reduce the defibrillation threshold, allowing for successful defibrillation of an otherwise refractory VF in ischemic as well as nonischemic substrates.
• Arrhythmic risk stratification after an acute myocarditis is complex and may require a different approach in the subacute phase compared with the chronic phase.

## Differential Diagnosis

First, an acute coronary syndrome had to be ruled out. The other potential differential diagnoses included acute myocarditis or the acute onset/manifestation of an inflammatory cardiomyopathy (hot phase).

## Investigations

Urgent coronary angiography was negative. A cardiac magnetic resonance imaging (CMR) scan was planned to investigate the possibility of acute myocarditis.

## Management

**Figure 1:**
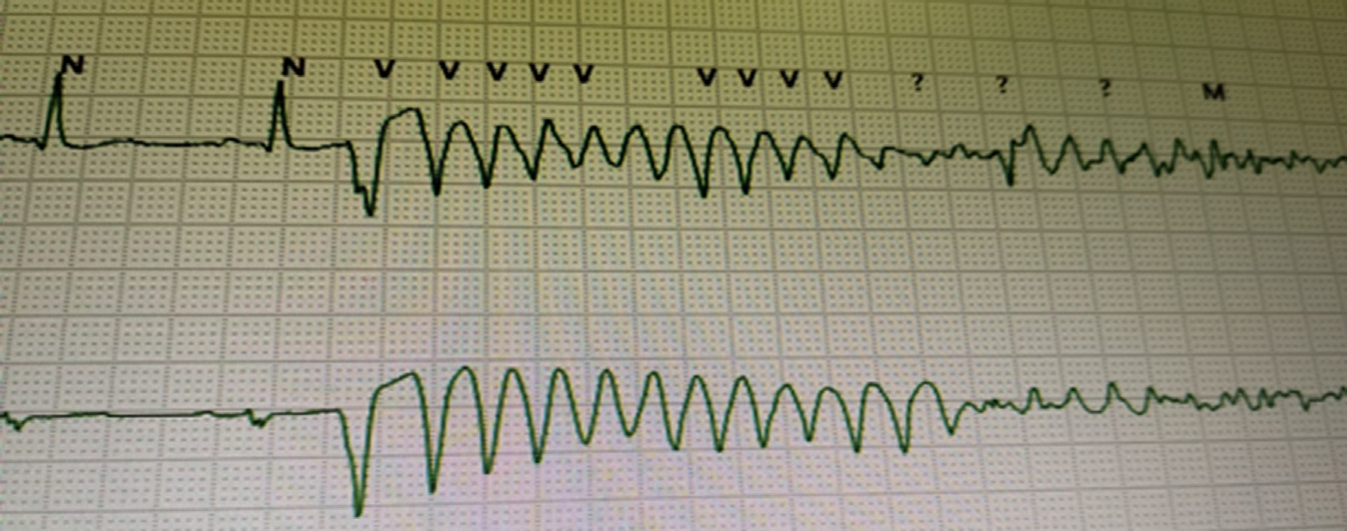
Polymorphic Ventricular Fibrillation Degenerating Into Ventricular Fibrillation

On day 2, 8 hours after presentation, despite a normal QT interval the patient began to experience frequent, isolated, premature ventricular complexes (PVCs) of left bundle branch block and superior axis morphology with an R-on-T phenomenon (coupling interval: 300 ms). A premature PVC led to a polymorphic ventricular tachycardia (pVT) that rapidly progressed into ventricular fibrillation (VF), which was successfully treated with a single DC shock (Figure 1). Lidocaine (100 mg bolus followed by continuous infusion at 20 μg/kg/min) and bisoprolol at 1.25 mg were started.

On day 2, 10 hours after presentation, the patient developed an electrical storm characterized by multiple episodes of pVT degenerating into VF and requiring multiple DC shocks (each one effective), a repeated lidocaine (100 mg) plus amiodarone bolus (300 mg), and 3 metoprolol boluses of 2 mg each. Regardless, the ventricular bigeminy persisted. The patient was deeply sedated, intubated, and transferred to our hub center.

Upon arrival at our hospital, on day 2 at 11:45 am, the patient experienced cardiac arrest due to a further episode of premature PVC-induced pVT degenerating into refractory VF. Advanced cardiovascular life support protocol was initiated, including the placement of a LUCAS (Lund University Cardiac Assist System). However, despite amiodarone and lidocaine bolus, 2 mg of MgSO_4_, 6 mg of adrenaline, and the initiation of venoarterial femoro-femoral extracorporeal membrane oxygenation (ECMO) (on day 2 at 12:20 pm), the patient remained in VF, which was unresponsive to multiple (n = 20) DC shocks attempts at 200 J, including double sequential and vector change defibrillation. Consequently, after an estimated total time of 52 minutes of ongoing VF, a percutaneous left stellate ganglion block (PLSGB) was performed using an ultrasound-guided approach with a local bolus of 150 mg of lidocaine 2% plus 50 mg of ropivacaine 1% (Table 1). After a 5-minute wait, a single DC shock was delivered that successfully restored a stable sinus rhythm at 35 beats/min.

**Table 1:** Equipment List

Ultrasound-guided Percutaneous Left Stellate Ganglion Block **IMAGING** • Vascular Echo Color Doppler (ECD) Philips CX50 o L12-3 linear probe (12-3 MHz) **Materials** • Surgical drapes • 20 mL sterile syringe (2 pieces) • Sterile gauzes pads • 0.9% sodium chloride solution vial (10 mL each, 2-3 pieces) • Spinal needle Quincke type point (22G × 3.00-inch, 0.7 × 75 mm) • Peripheral venous catheter (PVC) extension line (⌀ = 1 mm, L = 100 cm) • General purpose probe cover kit (probe cover 13 cm × 122 cm with gel pack) • Nonwoven adhesive medication (5 × 7 cm) **Drugs** • 100 mg of lidocaine hydrochloride 20 mg/mL (1 vial = 10 mL = 200 mg): use 5 mL (100 mg) • 100 mg of ropivacaine hydrochloride 10 mg/mL (1 vial = 10 mL = 100 mg): use 10 mL (100 mg) **TECHNIQUE** The patient underwent an ultrasonography-guided percutaneous left stellate ganglion block (PLSGB) at bedside during advanced cardiovascular life support including LUCAS (Lund University Cardiac Assist System). First, his head was turned to the opposite side. Chlorhexidine was used to prepare the anterior and lateral parts of the neck. A linear ultrasound probe was than applied to the anterolateral neck at the cricoid cartilage level to identify the transverse process of C6 and C7, anterior tubercle of C6 (Chassaignac tubercle), muscular structures (sternocleidomastoid muscle superficially, anterior scalene muscle laterally, and longus colli muscle in depth, anterior to the vertebral plane), and surrounding neurovascular structures (jugular vein and carotid artery medially, vertebral vessels laterally). Sodium chloride 0.9% was used to dissect planes and to help confirm the needle position. The local anesthetic solution was then injected beneath the prevertebral fascia and above the longus colli muscle.

Nonetheless, TTE demonstrated severe biventricular systolic dysfunction (LVEF 28%; tricuspid annular plane systolic excursion [TAPSE]: 11 mm), likely due to a combination of myocardial stunning after prolonged VF, bradycardia, and ongoing myocardial inflammation. In the subsequent hours, the patient developed oliguria, metabolic acidosis, and hepatic and renal impairment (creatinine: 3.16 mg/dL), which were effectively managed with vasopressin, dobutamine, and an HCO_3_^−^ infusion. An amiodarone drip (450 mg/day) was maintained to prevent further arrhythmic recurrences.

By day 3, significant improvement was observed, with an increase of biventricular contractility (LVEF 45%; left ventricular outflow tract velocity time integral: 16 cm; TAPSE: 20 mm). The patient was gradually weaned off the ECMO and vasopressors and was extubated. The myocarditis screening revealed serum enterovirus IgM positivity (without IgG) with negative autoimmunity; an endomyocardial biopsy (day 7) showed subendocardial fibrosis without lymphocytic inflammatory infiltrates in the analyzed sections of the right ventricle. Due to the mild polymorphism and cytoplasmic vacuolization of cardiomyocytes, combined with mild degenerative aspects at the ultrastructural analysis, genetic testing was performed to exclude an underlying arrhythmogenic cardiomyopathy.

**Figure 2:**
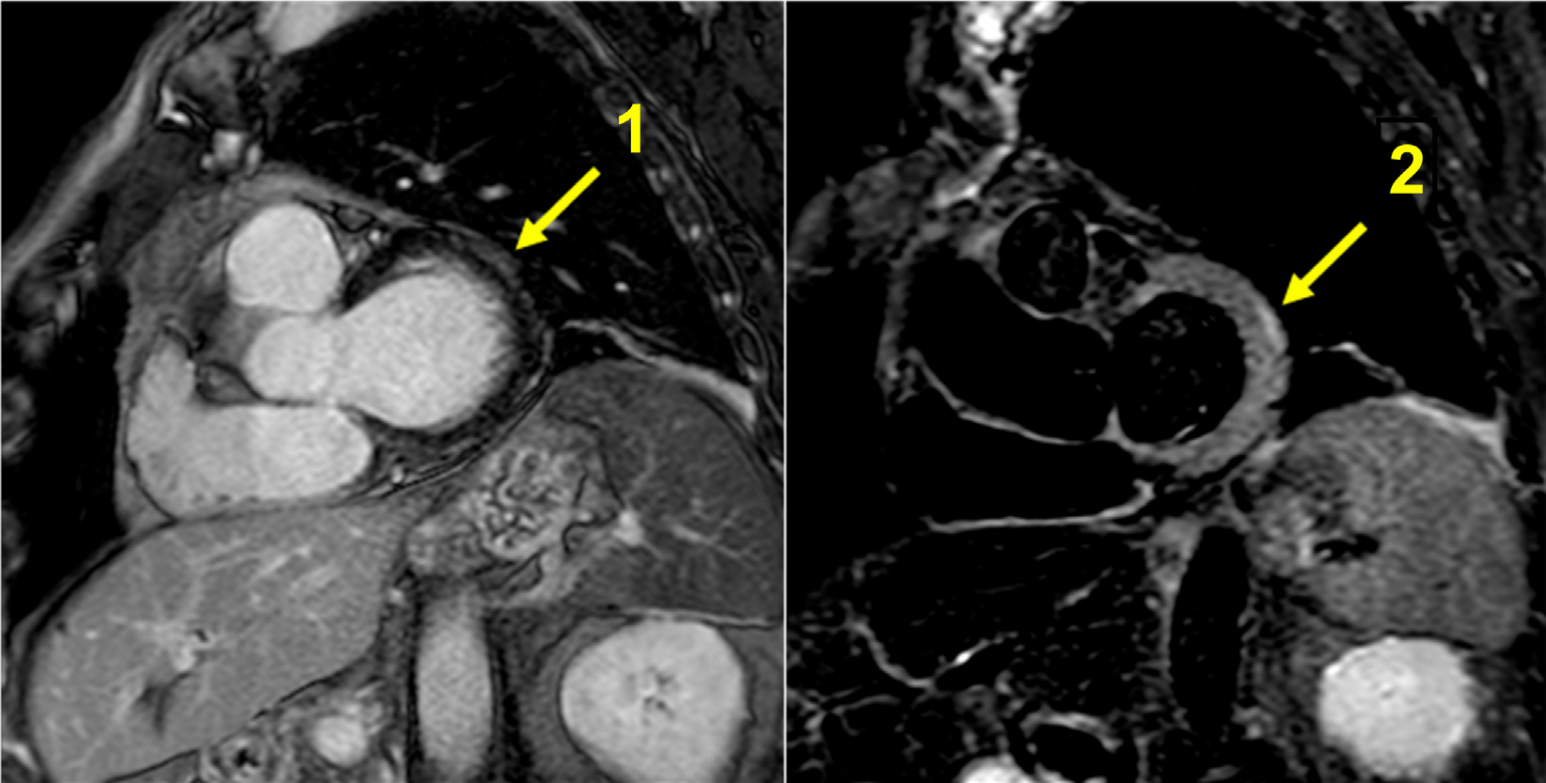
Cardiac Magnetic Resonance Late gadolinium enhancement at basal anterolateral and inferolateral subepicardial segment (nonischemic pattern) (1). Signal hyperintensity on T2 analysis at the basal anterolateral subepicardial segment (2).

Finally, CMR performed on day 11 (Figure 2) showed 50% LVEF, edema (signal hyperintensity on T2 analysis) at the subepicardial sites of the basal anterolateral left ventricular segment, and late gadolinium enhancement at the basal anterolateral and inferolateral subepicardial segments (nonischemic pattern), overall suggesting myocarditis (Lake Louise Criteria). Notably, the native T1 values and extracellular volume fraction mapping were within normal ranges.

In conclusion, a diagnosis of acute fulminant myocarditis was established. Fifteen days after admission, despite ongoing oral therapy with canrenone at 50 mg, amiodarone at 200 mg, and bisoprolol at 1.25 mg, the patient experienced a run of monomorphic ventricular tachycardia (right bundle branch block morphology with horizontal axis) with warm-up phenomenon (mean heart rate: 160 beats/min) lasting 16 seconds.

The temporary placement of a wearable defibrillator for 3-4 months followed by CMR reassessment was discussed. Due to issues of wearable defibrillator availability and the patient’s characteristics, we opted for a subcutaneous implantable cardioverter-defibrillator (ICD). The patient was discharged on day 21 on canrenone at 50 mg/day and nadolol at 40 mg/day (heart rate at discharge: 45 beats/min). The LVEF at TTE was 55%.

## Outcome and Follow-Up

The genetic testing (cardiomyopathy panel with next-generation sequencing, 176 genes) was negative. After 14 months, there were no symptomatic or treated VAs on nadolol at 30 mg/day (reduced due to bradycardia), with preserved biventricular function. Twenty-four hour Holter monitoring (minimum heart rate: 36 beats/min; mean: 60 beats/min; maximum: 111 beats/min) showed only 7 isolated PVCs (right bundle branch block morphology, superior axis) and 67 premature supraventricular complexes including 5 supraventricular couplets. Finally, only rare, isolated PVCs with no increase during effort were observed during the exercise stress test (maximum heart rate: 120 beats/min; 73% of the expected).

## Discussion

Acute fulminant myocarditis is an uncommon form of severe myocarditis, characterized by hemodynamic impairment and refractory VAs.^1^ The present clinical case highlights important learning and discussion points regarding acute and chronic arrhythmia management.

First, PLSGB usage has been typically reported to prevent VA recurrences in the setting of electrical storm due to its well-established antiarrhythmic effects acting on both the trigger and the substrate.^2^ In the largest study published so far on this topic, which included 131 patients,^3^ the benefit of PLSGB—measured by the number of VA episodes requiring treatment in the hour after compared with the hour before—was greater for patients with electrical storm due to recurrent VF than for those with either fast (cycle <375 ms) or slow (cycle ≥375 ms) ventricular tachycardias underlying the storm.^4^ Notably, acute coronary syndromes were the most common cause of VF-related electrical storm, and the timing of reperfusion, which was not specified, may have influenced the observed results.

In our case, PLSGB was performed while the patient had ongoing refractory VF in order to increase the probability of success of DC shock. Of note, preclinical studies have shown that PLSGB significantly increases the VF threshold,^5^ but it was never directly demonstrated that PLSGB performed during VF may improve defibrillation response. Few clinical cases have extensively reported so far on PLSGB allowing for successful refractory VF defibrillation;^6^ most have been in the setting of ST elevation myocardial infarction.^7^ Our case is the first to describe this in the setting of an acute myocarditis and thus in a completely different substrate.

Notably, advanced cardiovascular life support had been ongoing for more than 40 minutes before PLSGB. In this case, the choice was made to not administer steroids or intravenous immunoglobulin; although their usage before an endomyocardial biopsy is still controversial, they may be beneficial in stabilizing the rhythm and hemodynamic status even in cases of a lymphocytic form of acute fulminant myocarditis.^1^ Our main reasons were as follows: 1) the patient was already on ECMO due to refractory VF; 2) PLSGB had already effectively stabilized the rhythm; and 3) an endomyocardial biopsy, which should be performed before administering immunosuppressive drugs, had been delayed due to the patient’s overall improving clinical condition starting from the end of day 2.

Second, arrhythmic risk stratification after acute myocarditis remains controversial. The latest European guidelines advocate for ICD implantation as secondary prevention in patients who have experienced a hemodynamically not-tolerated VA during the acute phase of myocarditis;^8^ notably, the LVEF recovery and CMR patterns (presence and extension of late gadolinium enhancement and edema), which might be more tightly associated with the future risk of VAs, are not considered.^9^^,^^10^ Additionally, some transient proarrhythmogenic triggers may diminish over time, rendering the indication for predischarge ICD implantation controversial. Bridge therapy with a wearable ICD could be a potential solution for selected patients, but issues regarding accessibility and the optimal duration of such treatment remain unresolved.

**Visual Summary:**
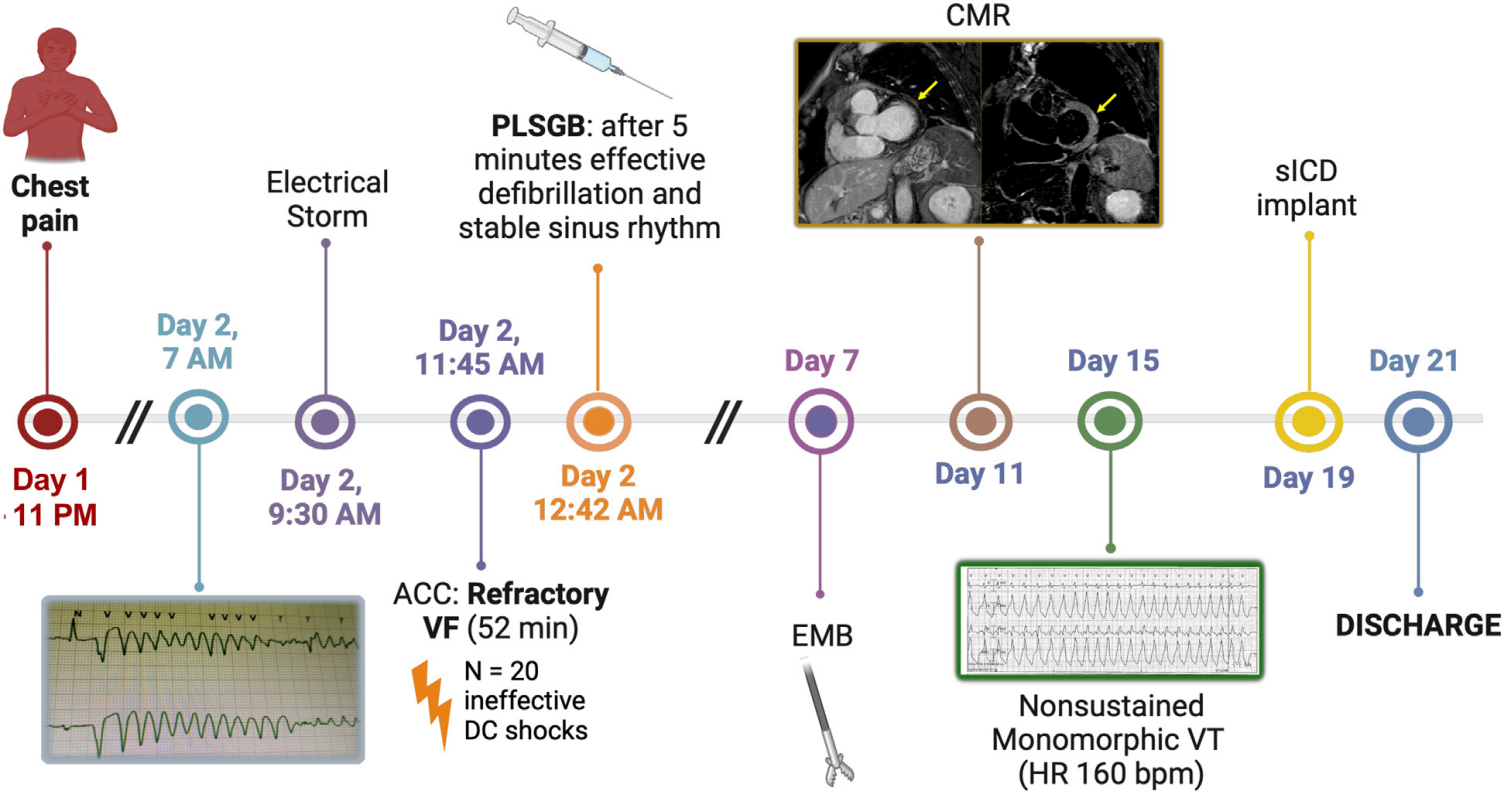
Case Report Timeline bpm = beats/min; CMR = cardiac magnetic resonance; EMB = endomyocardial biopsy; HR = heart rate; PLSGB = percutaneous left stellate ganglion block; pVT = polymorphic ventricular tachycardia; sICD = subcutaneous implantable cardioverter defibrillator; VF = ventricular fibrillation; VT = ventricular tachycardia.

## Conclusions

PLSGB played a key role for the acute management of a premature PVC-induced pVT degenerating into refractory VF in the setting of acute fulminant myocarditis, allowing for acute stabilization of the patient. More clinical data are needed on the usage of acute neuromodulation to improve defibrillation response in the setting of refractory cardiac arrest and on arrhythmic risk stratification after myocarditis.

## Funding Support and Author Disclosures

The authors have reported that they have no relationships relevant to the contents of this paper to disclose.
